# Does the social context of early alcohol use affect risky drinking in adolescents? Prospective cohort study

**DOI:** 10.1186/s12889-015-2443-5

**Published:** 2015-11-16

**Authors:** Louisa Degenhardt, Helena Romaniuk, Carolyn Coffey, Wayne D. Hall, Wendy Swift, John B. Carlin, Christina O’Loughlin, George C. Patton

**Affiliations:** National Drug and Alcohol Research Centre, UNSW Australia, Sydney, NSW 2052 Australia; Centre for Adolescent Health, Murdoch Children’s Research Institute, Melbourne, Australia; School of Population and Global Health, University of Melbourne, Melbourne, Australia; Department of Global Health, School of Public Health, University of Washington, Seattle, USA; Clinical Epidemiology and Biostatistics Unit, Murdoch Children’s Research Institute, Melbourne, Australia; Department of Paediatrics, University of Melbourne, Melbourne, Australia; Centre for Youth Substance Abuse Research, University of Queensland, Brisbane, Australia; King’s College London (Institute of Psychiatry), King’s College London, London, UK

**Keywords:** Alcohol, Adolescence, Risky drinking, Binge drinking

## Abstract

**Background:**

There are limited longitudinal data on the associations between different social contexts of alcohol use and risky adolescent drinking.

**Methods:**

Australian prospective longitudinal cohort of 1943 adolescents with 6 assessment waves at ages 14–17 years. Drinkers were asked where and how frequently they drank. Contexts were: at home with family, at home alone, at a party with friends, in a park/car, or at a bar/nightclub. The outcomes were prevalence and incidence of risky drinking (≥5 standard drinks (10g alcohol) on a day, past week) and very risky drinking (>20 standard drinks for males and >11 for females) in early (waves 1–2) and late (waves 3–6) adolescence.

**Results:**

Forty-four percent (95 % CI: 41-46 %) reported past-week risky drinking on at least one wave during adolescence (waves 1–6). Drinking at a party was the most common repeated drinking context in early adolescence (28 %, 95 % CI 26-30 %); 15 % reported drinking repeatedly (3+ times) with their family in early adolescence (95 % CI: 14-17 %). For all contexts (including drinking with family), drinking 3+ times in a given context was associated with increased the risk of risky drinking in later adolescence. These effects remained apparent after adjustment for potential confounders (e.g. for drinking with family, adjusted RR 1.9; 95 % CI: 1.5-2.4). Similar patterns were observed for very risky drinking.

**Conclusions:**

Our results suggest that consumption with family does not protect against risky drinking. Furthermore, parents who wish to minimise high risk drinking by their adolescent children might also limit their children’s opportunities to consume alcohol in unsupervised settings.

**Electronic supplementary material:**

The online version of this article (doi:10.1186/s12889-015-2443-5) contains supplementary material, which is available to authorized users.

## Background

Considerable research has examined the place of alcohol in different societies and cultures [1]. There has also been particular interest in the possibility that the social and cultural context in which drinking is initiated affects later patterns of alcohol consumption [2]. This idea underpins a strategy of introducing adolescents to alcohol in the family home under parental supervision to encourage less risky patterns of alcohol consumption.

Yet the evidence on whether the social context of drinking, including consumption with family, affects adolescents’ likelihood of engaging in risky drinking is unclear. The few studies of associations between adolescent consumption of alcohol with parents, and patterns of alcohol consumption in adolescents, provide conflicting evidence [2, 3]. Two found that there were higher levels of adolescent consumption of alcohol among those who were provided alcohol by their parents compared to those who were not [4]. Other studies have found that adolescents who report drinking with parents, compared to those who do not report drinking with their parents, have lower levels of risky drinking [5–8] but a higher frequency of alcohol consumption [9–12]. More recent studies suggest that adult-supervised settings for alcohol use may actually *increase* risks of harmful use [13]; and that the more available alcohol is in the home, the lower the age of alcohol initiation [14]. However, all these studies have been cross-sectional and so have been unable to determine any direction of effect.

There has been limited examination of other drinking settings, particularly ones with less parental supervision, such as parties. It may well be that consuming alcohol in less supervised settings than the family home increases the risks for heavier alcohol use. Adolescents who drink with their parents may drink more either because they are already drinking and hence more likely to drink in a range of different contexts, or because drinking at home increases risky drinking. As a consequence, it is unclear what advice we should give parents about when and how to introduce their children to alcohol [2]. Some have suggested that parents should promote a drinking culture in which alcohol is used in moderation. Others fear that an early introduction to alcohol may normalise adolescent alcohol use and increase the chances of harmful use [15].

This paper examines predictive associations between the contexts in which alcohol is consumed in early adolescence and risky [16] (also termed “binge”) drinking in later adolescence, in an established Australian longitudinal study of adolescent development: the Victorian Adolescent Health Cohort Study. It assesses these associations after adjusting for the effects of known risk factors for risky alcohol use, such as parental alcohol consumption, and adolescent mental health and other risk behaviours. Specifically, we examined:

1. The prevalence and incidence of adolescent risky drinking (5 or more standard drinks (each 10 g alcohol) in a session) and very risky drinking (>20 standard drinks for males and >11 standard drinks for females in a session);
2. The frequency of drinking in different contexts reported in early adolescence, including parental supply and supervision;
3. Predictive associations between the contexts in which alcohol is used in early adolescence and incident risky drinking and very risky drinking in later adolescence.

## Methods

### Sample

Between August 1992 and January 2008 we conducted a nine-wave cohort study of health in young people living in the state of Victoria, Australia. The Ethics in Human Research Committee of the Royal Melbourne Children’s Hospital approved all data collection protocols. Informed written parental consent was obtained before inviting students to participate.

**Fig. 1:**
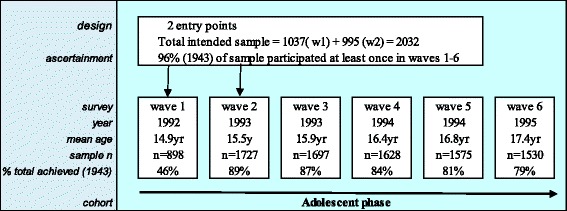
Design, sampling and ascertainment in the Victorian Adolescent Health Cohort

A two-stage cluster sampling procedure, first by school and then by class, was used to select the study population with class recruitment at two entry points. At stage one, 45 schools were randomly chosen from a stratified frame of government, Catholic and independent private schools with the probability of selection in each stratum proportional to the number of students of that age in each school. At stage two of the sampling procedure there were two entry points, with one intact class selected from each participating school in wave 1, and a second class selected six months later (wave 2). These two classes from each participating school then formed the basis of the cohort which was followed up during adolescence a further 4 times at six monthly intervals (waves 3 to 6) and during young adulthood 3 times (waves 7 to 9) (Fig. 1). After wave 1, one school (*n* = 13 participants) declined continued participation, leaving 44 study schools.

The analysis in this paper is mainly based on data collected during the adolescent waves (waves 1 to 6), with adult wave data (waves 7 and 8) only used to identify parental divorce and separation occurring during adolescence that was reported retrospectively by study participants in adulthood. From a total sample of 2032 invited participants, 1943 (96 %) participated at least once during the six adolescent waves. Seventy six invited participants were either refused consent by their parents or were never available for interview (Fig. 1). Participants self-administered the questionnaire on laptop computers, with telephone follow-up for those who were absent from school after wave 2. The seventh to ninth waves were undertaken using computer-assisted telephone interviews.

### Alcohol measures (at each wave)

#### Drinking contexts

In Australia, the legal age for purchase of alcohol is 18 years. Consumption of alcohol by those under 18 years is illegal, unless under the direct supervision of a parent or another adult acting in *loco parentis*. All participants except those who said they were a non-drinker were asked where they drank and whether they did so repeatedly in the last 6 months (never, once or twice, less than monthly, or more than monthly). The drinking contexts were: at home with family, at home alone, at a party with friends, at a pub or place to eat, in a park or on the street or at the beach, in a car, or at a nightclub.

Within each wave of data collection and for each drinking context, we identified adolescents who (a) never drank in that setting, and for those who did so, we categorised frequency of drinking in that context as occurring (b) 1–2 times for those reporting “once or twice” or (c) 3+ times for those reporting “less than monthly” or “more than monthly”. We then combined, firstly, “in a park or on the street or at the beach” with “in a car” into a single measure termed “in a park/car”, and, secondly, “at a pub or place to eat” with “at a nightclub” into a single measure “in a bar/club”. For the combined categories, drinking was categorised as 3+ times if adolescents reported drinking “less than monthly” in both contexts or “more than monthly” in at least one context. In early adolescence (waves 1 & 2), for each context we identified those adolescents who were drinking 3+ times at one or both waves, and termed this “repeated” use in that context in early adolescence; and identified those with non-repeated use as non-drinkers or those who were drinking less than 3 times in that context at both waves.

#### Alcohol consumption

Participants who reported that they drank alcohol in the week prior to interview were asked to complete a beverage- and quantity-specific one-week alcohol diary (for more details see [17]). We calculated the number of alcohol units (1 unit = 10 g of alcohol) consumed each day of the diary week. Risky drinking was defined as having drunk 5 or more units on at least one day during the week prior to survey. Very risky drinking was defined as having drunk >20 units for males and >11 units for females on any day over the diary week [16, 18]. Incident risky and very risky drinking were identified in late adolescence.

### Time-varying measures assessed in each wave

#### Daily tobacco use

We identified participants using tobacco daily.

#### Regular cannabis use

We identified participants using cannabis ≥ weekly.

*Antisocial behaviour* was assessed using 10 items from the Moffitt and Silva self-report early delinquency scale [19], assessing property damage, interpersonal conflict and theft. Items concerning alcohol use or other substance use were not included. Participants were asked if they had engaged in any of these behaviours *never, once,* or *more than once* in the last 6 months*.* At each wave, antisocial behaviour was identified in participants who reported multiple behaviours at least once or one behaviour more than once.

*Mental health* was assessed using the Clinical Interview Schedule (CIS-R) [20], which assesses symptoms of depression and anxiety in non-clinical populations. We identified those participants with a score >11 as having a mixed depression-anxiety state for which clinical intervention would be appropriate.

### Background measures

#### Geographic location

Rural and urban areas were defined using the location of the school at study inception.

*Frequency of parental drinking and smoking* was assessed in the course of the study. Regular parental smoking and drinking was defined as at least one parent engaging in these behaviours on most or every day.

*Parental divorce or separation* in adolescence (by wave 6) was identified in the course of the adolescent surveys or from responses to enquiry about parental marital status in later waves if the adolescent was absent at wave 6.

### Statistical analysis

Prevalence and incidence of risky drinking and very risky drinking were estimated overall and separately for males and females at each wave within a multiple imputation framework (see below). The frequency of drinking in each of the drinking contexts was estimated for males and females in early adolescence (waves 1 & 2). The association between drinking with family at home and drinking in each other context in early adolescence was estimated using odds ratios.

The association between adolescent drinking contexts measured at waves 1 and 2 and incident risky drinking in late adolescence (waves 3–6) was assessed using repeated measures discrete time proportional hazards models [21]. Adjustment was initially for (i) wave of observation and then for (ii) background factors (sex, school location (urban or rural) and parental divorce/separation, parental frequent alcohol use and parental cigarette smoking) and time varying adolescent measures at the previous wave (daily cigarette smoking, weekly + cannabis use, antisocial behaviour and symptoms of anxiety and depression). Initially, the effect of drinking with family and each *other* drinking context (i.e. home alone, at pub/club, at a party, in a park/car) was estimated separately and then jointly to assess the effect of each drinking context on incident risky drinking. To estimate the effect of joint drinking locations, we generated new four level variables for each *other* context: not drinking in *other* location and not drinking at home with family (baseline category); not drinking in *other* location and drinking with family; drinking in *other* location and not drinking with family; drinking in *other* location and with family.

In these models, wave of observation was entered as three dummy variables with wave 3 as reference category in order to avoid constraining the effect of wave/time to be linear. Interactions between sex and drinking context, and sex and wave were assessed in the fully adjusted separate models. There was no evidence of interactions between any of these variables so they were not retained in the final models. All main effects and interactions were tested for statistical significance using Wald tests . All data analysis was conducted using Stata 13 [22].

Multiple imputation [23] was used to handle missing data. We imputed 20 complete datasets, separately for males and females, under a multivariate normal model that incorporated all variables used in the analysis and auxiliary variables that were thought to be related to missingness. The imputation model contained 36 key variables used in the analysis and 29 auxiliary variables not used in the analysis but thought to be related to the missingness. Auxiliary variables included in the imputation model were age at wave 2, context of drinking at waves 3–6 and adolescent measures (smoking, cannabis use, antisocial behavior and symptoms of anxiety and depression at waves 1 and 6. Of these 65 variables, five had <10 % missing values, 19 had 10–14.9 % missing and 16 had 15–19.9 % missing, 15 had 20-23 % missing and 10 wave 1 variables had 53-61 % missing (because about half the cohort was not recruited until wave 2). A three level maximum drinking variable was created at each wave: no risky drinking, risky drinking and very risky drinking. These variables and context of drinking variables were log transformed before imputation. All other variables were not transformed before imputation.

After imputation, any transformed variables were converted back to their original scale and categorised for analysis, with adaptive rounding for binary measures [24]. Simple diagnostics were used [25] to assess if the imputed datasets were reasonable. The imputed distributions were similar to the distributions of observed values for all variables. All estimates were obtained by averaging results across the twenty imputed datasets with inferences under multiple imputation obtained using Rubin’s rules [23].

## Results

Fifty one percent of the sample was female, 26 % attended a rural school at study inception, 39 % reported that at least one parent smoked cigarettes on most days, 32 % reported that their parent drank alcohol on most days, and 23 % reported that their parents were separated or divorced by wave 6.

Overall, 44 % (95 % CI: 41-46 %) of participants reported risky drinking in the past week on at least one wave during adolescence. This was more common in males (53 %, 95 % CI: 49-57 %) than females (35 %, 95 % CI 32-39 %). The frequency of risky drinking in the past week increased steadily across adolescence: from 7 % in wave 1 to 23 % in wave 6 (Table 1).

**Table 1:** Estimates of the prevalence of risky drinking in the week prior to survey by sex, wave and phase

Risky drinking	Male			Female			Total		
	(*N* = 943)			(*N* = 1000)			(*N* = 1943)		
	Number	Percentage	(95 % CI)	Number	Percentage	(95 % CI)	Number	Percentage	(95 % CI)
by wave									
Wave 1 (mean age 14.9 years)	81	9	(6–11)	62	6	(4–8)	143	7	(6–9)
Wave 2 (mean age 15.4 years)	149	16	(13–18)	78	8	(6–10)	227	12	(10–13)
Wave 3 (mean age 15.9 years)	174	18	(16–21)	103	10	(8–12)	277	14	(13–16)
Wave 4 (mean age 16.3 years)	212	22	(20–25)	140	14	(12–16)	352	18	(16–20)
Wave 5 (mean age 16.8 years)	267	28	(25–32)	152	15	(13–18)	419	22	(19–24)
Wave 6 (mean age 17.4 years)	298	32	(28–35)	151	15	(13–17)	449	23	(21–25)
by phase									
In early adolescence (waves 1&2)	186	20	(17–23)	111	11	(9–13)	296	15	(13–17)
In late adolescence (waves 3–6)	470	50	(46–53)	324	32	(29–36)	794	41	(39–43)
In adolescence (waves 1–6)	499	53	(49–57)	354	35	(32–39)	853	44	(41–46)

Results for very risky drinking are provided in detail in the Additional file 1. Around one in five participants reported very risky drinking on at least one wave during adolescence. Similar levels were reported by males (20 %, 95 % CI: 17-23 %) and females (16 %, 95 % CI 13-18 %). The frequency of very risky drinking also increased steadily across adolescence: from 1 % at wave 1 to 6 % at wave 6 (Additional file 1: Table S1).

At each wave in late adolescence, 8-11 % of adolescents reported incident risky drinking and 3-5 % of adolescents reported incident very risky drinking. Incident levels at each wave were similar for males and females for both levels of risky drinking (Tables 2, Additional file 1: Table S2).

**Table 2:** Estimates of incident risky drinking in late adolescence by wave and sex

Incident risky drinking	Male				Female					Total		
	Total^a^	Number^b^	Percentage	(95 % CI)	Total^a^	Number^b^	Percentage	(95 % CI)	Total^a^	Number^b^	Percentage	(95 % CI)
Wave 3 (mean age 15.9 years)	757	77	10	(8–13)	889	56	6	(5–8)	1647	134	8	(7–10)
Wave 4 (mean age 16.3 years)	680	90	13	(10–16)	833	75	9	(7–11)	1513	165	11	(9–13)
Wave 5 (mean age 16.8 years)	591	76	13	(10–16)	758	53	7	(5–9)	1348	129	10	(8–11)
Wave 6 (mean age 17.4 years)	515	71	14	(10–17)	705	58	8	(6–11)	1220	130	11	(9–13)

^a^Total number of adolescents seen at each wave with no previous occurrence of risky drinking

^b^Number of adolescents with incident risky drinking at wave

**Fig. 2:**
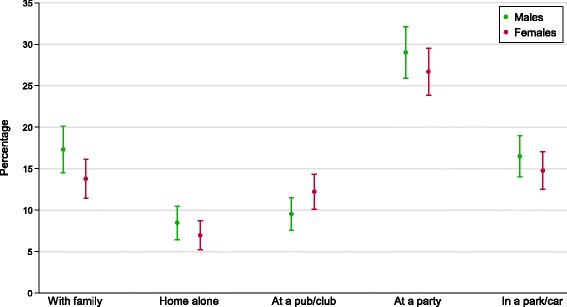
Proportion of participants reporting repeated (3+ times) drinking in each context in early adolescence (waves 1 & 2) by sex, with 95 % confidence intervals. Repeated drinking refers to 3+ occasions in the past 6 months, and is relative to not drinking in that context or drinking in that context on only 1 or 2 occasions

During early adolescence, 37 % (95 % CI 35-40 %) of adolescents reported drinking repeatedly (three or more times in the past six months) in at least one context. Slightly more males (40 %, 95 % CI 37-44 %) than females (34 %, 95 % CI 31-38 %) reported drinking 3+ times in the past 6 months in one or more contexts in early adolescence. Figure 2 shows the context that participants reported drinking 3 or more times in the past 6 months during early adolescence, by sex (Additional file 1: Table S3 provides full details). Males and females reported similar levels of repeated drinking in each context during early adolescence. Fifteen percent of participants reported drinking repeatedly with their family in early adolescence (95 % CI 14-17 %). Drinking at a party was the most commonly reported repeated drinking context in early adolescence (28 %, 95 % CI 26-30 %), and drinking alone the least common (8 %, 95 % CI 6-9 %).

**Fig. 3:**
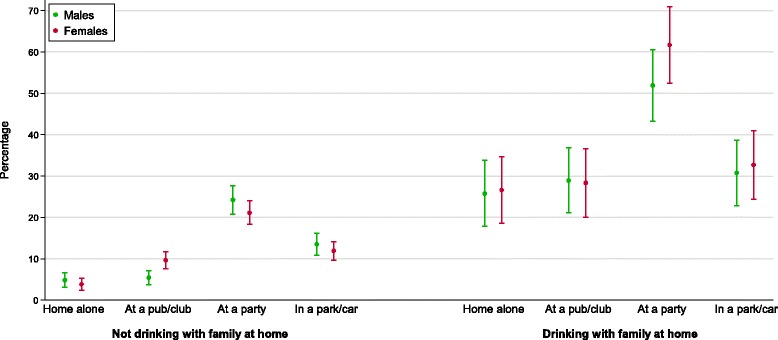
Proportion of participants reporting repeated drinking in each context in early adolescence (waves 1 & 2) by family context and sex, with 95 % confidence intervals. Repeated drinking refers to 3+ occasions in the past 6 months, and is relative to not drinking in that context or drinking in that context on only 1 or 2 occasions

During early adolescence, males and females who reported repeated drinking at home with family were more likely to report repeated drinking in other contexts than those who did not drink repeatedly at home with their parents (Fig. 3). Males who reported repeatedly drinking with family were more likely to repeatedly drink alone (OR 6.8, 95 % CI 4.0-11.7), in a pub or club (OR 7.0, 95 % CI 4.2-11.8), at a party (OR 3.4, 95 % CI 2.3-5.0) and in a park or car (OR 2.8, 95 % CI 1.8-4.5) than males who did not repeatedly drink with family. Females who reported repeatedly drinking with family were also more likely to repeatedly drink alone (OR 9.1, 95 % CI 5.2-16.2), in a pub or club (OR 3.7, 95 % CI 2.3-5.9), at a party (OR 6.0, 95 % CI 3.9-9.3) and in a park or car (OR 3.6, 95 % CI 2.3-5.5) than females who did not drink repeatedly at home with family.

Table 3 reports the associations between repeated drinking in different contexts in early adolescence and incident risky drinking in late adolescence. Adolescents who drank 3+ times in each context were more likely to report risky drinking in later adolescence when modelled separately adjusting only for wave of observation. These effects remained after adjustment for other factors known to be associated with risky drinking (e.g. antisocial behaviour, other substance use). These associations did not differ by sex or by wave. A similar pattern was found for associations between repeated drinking in each of the different contexts in early adolescence and incident very risky drinking in late adolescence (Additional file 1: Table S4).

**Table 3:** Association between context of drinking in early adolescence and incident risky drinking in later adolescence

				Incidence over waves 3 to 6 (*N* = 1647)					
		Number drinking in each context^b^		Number incident		Model estimates^a^			
				Risky drinking		Unadjusted^c^		Adjusted^d^	
Drinking context		Number	(%)	Number	(%)	RR	(95 % CI)	RR	(95 % CI)
With family	*no*	1437	(87)	441	(31)	1		1	
	*yes*	209	(13)	115	(55)	2.2	(1.8 – 2.8)	1.9	(1.5 – 2.4)
Home alone	*no*	1577	(96)	507	(32)	1		1	
	*yes*	70	(4)	50	(71)	3.3	(2.4 – 4.6)	2.4	(1.6 – 3.6)
At a pub/club	*no*	1550	(94)	494	(32)	1		1	
	*yes*	97	(6)	63	(65)	2.9	(2.1 – 4.0)	2.5	(1.7 – 3.5)
At a party	*no*	1338	(81)	354	(26)	1		1	
	*yes*	308	(19)	203	(66)	3.7	(3.0 – 4.5)	2.9	(2.3 – 3.7)
In a park/car	*no*	1510	(92)	459	(30)	1		1	
	*yes*	137	(8)	98	(71)	3.8	(2.9 – 5.0)	2.5	(1.9 – 3.4)

^a^Separate model fitted for each drinking context

^b^Prevalence for those who had no risky drinking in waves 1 & 2

^c^Relative risks from multivariable discrete time proportional hazards models adjusted for wave of observation of risky drinking

^d^Relative risks from multivariable discrete time proportional hazards models adjusted for: (a) the wave of observation of risky drinking, (b) background measures: sex, rural school location, parental separation/divorce, parental frequent alcohol use and parental cigarette smoking (c) adolescent measures from the previous wave: daily cigarette smoking, weekly + cannabis use, antisocial behaviour and symptoms of anxiety and depression

Table 4 reports the joint associations between drinking with family and each *other* context, with incident risky drinking in late adolescence. In each case, adolescents who drank 3+ times in either or both contexts were more likely to report risky drinking in later adolescence than those who drank in neither context. Drinking with family also appeared to increase the risk of incident risky drinking for those drinking in each of the *other* contexts compared to those just drinking with family. These effects remained after adjusting for other factors known to influence risky drinking. The same pattern was found for incident very risky drinking (Additional file 1: Table S5).

**Table 4:** Association between repeated drinking in joint contexts in early adolescence and incident risky drinking in later adolescence

Joint drinking contexts in early adolescence (waves 1 & 2)				Incidence over waves 3 to 6 (*N* = 1647)					
		Number drinking in each joint context^b^		Number incident		Model estimates^a^			
				Risky drinking		Unadjusted^c^		Adjusted^d^	
Non family context	Family context	Number	(%)	N	(%)	RR	(95% CI)	RR	(95% CI)
Not home alone	Not with family	1402	(85)	418	(30)	1		1	
Not home alone	With family	175	(11)	89	(51)	2.0	(1.6 – 2.6)	1.8	(1.3 – 2.3)
Home alone	Not with family	36	(2)	23	(66)	3.2	(2.0 – 5.1)	2.1	(1.3 – 3.6)
Home alone	With family	35	(2)	26	(76)	4.2	(2.6 – 6.8)	3.3	(1.8 – 5.9)
Not at a party	Not with family	1219	(74)	301	(25)	1		1	
Not at a party	With family	119	(7)	53	(45)	2.1	(1.5 – 2.8)	1.8	(1.3 – 2.5)
At a party	Not with family	218	(13)	141	(65)	3.8	(3.0 – 4.8)	2.9	(2.2 – 3.7)
At a party	With family	90	(5)	62	(69)	4.4	(3.1 – 6.3)	3.8	(2.6 – 5.5)
Not at pub/club	Not with family	1374	(83)	397	(29)	1		1	
Not at pub/club	With family	175	(11)	97	(55)	2.4	(1.9 – 3.0)	2.1	(1.6 – 2.7)
At pub/club	Not with family	63	(4)	45	(71)	3.8	(2.7 – 5.4)	3.2	(2.2 – 4.7)
At pub/club	With family	34	(2)	18	(53)	2.4	(1.3 – 4.5)	2.0	(1.0 – 4.0)
Not in park/car	Not with family	1337	(81)	373	(28)	1		1	
Not in park/car	With family	173	(11)	86	(50)	2.1	(1.6 – 2.7)	1.8	(1.4 – 2.4)
In park/car	Not with family	101	(6)	69	(68)	3.8	(2.8 – 5.2)	2.4	(1.7 – 3.5)
In park/car	With family	36	(2)	29	(80)	5.8	(3.5 – 9.6)	4.2	(2.4 – 7.5)
Not in any other context	Not with family	1177	(71)	279	(24)	1		1	
Not in any other context	With family	100	(6)	46	(46)	2.2	(1.6 – 3.1)	1.9	(1.3 – 2.7)
In at least one other context	Not with family	261	(16)	163	(62)	3.8	(3.0 – 4.7)	2.9	(2.2 – 3.7)
In at least one other context	With family	109	(7)	69	(64)	4.0	(2.9 – 5.6)	3.4	(2.4 – 4.8)

Repeated drinking repeated refers to 3+ occasions in the past 6 months, and is relative to not drinking in that context or drinking in that context on only 1 or 2 occasions

^a^Separate model fitted for each joint drinking context

^b^Prevalence for those who had no risky drinking in waves 1 & 2

^c^Relative risks from multivariable discrete time proportional hazards models adjusted for wave of observation of risky drinking

^d^Relative risks from multivariable discrete time proportional hazards models adjusted for: (a) the wave of observation of risky drinking, (b) background measures: sex, rural school location, parental separation/divorce, parental frequent alcohol use and parental cigarette smoking (c) adolescent measures from the previous wave: daily cigarette smoking, weekly + cannabis use, antisocial behaviour and symptoms of anxiety and depression

## Discussion

We did not find evidence that drinking with family was protective against future adolescent risky drinking. Indeed, we found a substantial association in the opposite direction in that the onset of risky drinking was more likely among young people who had consumed alcohol with family on multiple occasions (3+ in the previous 6 months) than among those who had done so less frequently, or not at all. Incident risky drinking was also even more likely among young people who reported drinking in contexts where they had limited (or no) parental or adult supervision (parties, pubs, clubs, parks and cars).

To date, the risks of varied social contexts of alcohol use have not been examined in a prospective study, while taking account of confounding. Our results challenge the rationale for parents supervising alcohol consumption by adolescents as a strategy to reduce harmful drinking. Drinking in less supervised contexts also increased risks for later risky drinking.

It is difficult, based on observational data, to infer that the associations we observed are causal. For example, the consumption of alcohol with family may be hypothesised to reflect permissive parental attitudes towards drinking [26]. These associations nonetheless remained apparent after controlling for parental regular alcohol use (which is associated with attitudes), providing little support for this hypothesis. An additional possibility is that early adolescent drinking in *any* context, including with parents, heightens risks for later risky patterns of alcohol use. This would be consistent with evidence that earlier onset alcohol use is an important predictor of later problematic alcohol use [27] and that adolescence is a critical period during which exposure to alcohol carries particular risks for the development of later risky consumption patterns [28].

Such a possibility is consistent with a Dutch cohort study of adolescents, where it was found that drinking in the home was not protective against later “problem” drinking [29], and specifically that drinking with parents was also not protective – it did not matter with whom the adolescents were drinking, as all consumption increased odds of later problem drinking [29]. In that study, among mid-adolescent boys, drinking with parents also increased the odds of later drinking *outside* the home, which additionally increased risks of “problem” drinking [29]. This possibility would suggest that delaying the onset of drinking in general might be preferable as a strategy to reduce later risky alcohol use, rather than using a strategy where parents introduce their teenage children to alcohol [29].

### Limitations

This study has the strengths of prospective design and multiple waves of measurement but we did not ascertain the quantity of alcohol consumed in each of the social contexts. Further, our data may not have captured the full extent of risky drinking in this cohort because not all risky alcohol use will have occurred within the one week reference period. Both of these limitations mean that our estimated levels of risky drinking probably underestimate the total amount of risky drinking at each wave, and over time. All data were also based on self-report, but there is reasonable evidence that young people’s estimation of their alcohol use are reasonably reliable and valid when these reports are made in a confidential manner without any consequences for disclosing use, as was the case here [30, 31].

These findings may not apply in all cultural settings [1]. In Mediterranean countries (e.g. Italy, Greece, France), for example, alcohol use tends to be integrated into everyday life, smaller amounts of alcohol are more often consumed with meals, and youth report significantly fewer risky drinking episodes than youth in Anglophone countries such as Australia [32]. It is unclear whether less supervised alcohol consumption in these cultures would increase the risks of risky drinking.

Similarly, it is also unclear to what extent these findings might apply to countries with zero-tolerance policies towards drinking in adolescence (e.g. USA). However, recent work in the United States has suggested that, as we found in this study, the most common social contexts of alcohol use among adolescents were at a party or at home, and most commonly with one or two other people [33], suggesting that at least in terms of the context of drinking, it appears to be similar in that country.

## Conclusions

A significant proportion of the Australian adolescents we studied engaged in risky drinking during adolescence, and they drank alcohol in a variety of contexts. It does not seem that early adolescent drinking with the family was protective against later risky drinking. Parents wishing to reduce their children’s alcohol use may be better advised to limit the opportunities for adolescents to drink alcohol in both supervised and unsupervised settings.

## Additional file

Additional file 1: Table S1. Estimates of the prevalence of very risky drinking in the week prior to survey by sex, wave and phase. **Table S2.** Estimates of incident very risky drinking in late adolescence by wave and sex. **Table S3.** Estimates of the prevalence of repeated (3+ times) drinking in each context in early adolescence by sex. **Table S4.** Association between context of drinking in early adolescence and incident very risky drinking in later adolescence. **Table S5.** Association between joint context of repeated drinking in early adolescence and incident very risky drinking in later adolescence. (DOCX 42 kb)
