# Supplementary material for: Does the social context of early alcohol use affect risky drinking in adolescents? Prospective cohort study
Source: BMC Public Health. 2015 Nov 16;15:1137. doi: 10.1186/s12889-015-2443-5 (PMC4647618; doi:10.1186/s12889-015-2443-5)
Supplement: Additional file 1: Table S1. — Estimates of the prevalence of very risky drinking in the week prior to survey by sex, wave and phase. Table S2. Estimates of incident very risky drinking in late adolescence by wave and sex. Table S3. Estimates of the prevalence of repeated (3+ times) drinking in each context in early adolescence by sex. Table S4. Association between context of drinking in early adolescence and incident very risky drinking in later adolescence. Table S5. Association between joint context of repeated drinking in early adolescence and incident very risky drinking in later adolescence. (DOCX 42 kb) [file 12889_2015_2443_MOESM1_ESM.docx]

**Additional file 1**

**Table S1: Estimates of the prevalence of very risky drinking in the week prior to survey by sex, wave and phase**

| **Very risky drinking** |  | **Male**  **(N=943)** | | | |  | **Female**  **(N=1000)** | | | |  | **Total**  **(N=1943)** | | | |
| --- | --- | --- | --- | --- | --- | --- | --- | --- | --- | --- | --- | --- | --- | --- | --- |
|  |  | **Number** | | **%** | **(95%CI)** |  | **Number** | | **%** | **(95%CI)** |  | **Number** | | **%** | **(95%CI)** |
| **by wave** |  |  |  |  |  |  |  |  |  |  |  |  |  |  |  |
| Wave 1 (mean age 14.9 years) |  | 12 |  | 1 | (1 - 2) |  | 10 |  | 1 | (0 - 2) |  | 22 |  | 1 | (1 - 2) |
| Wave 2 (mean age 15.4 years) |  | 43 |  | 5 | (3 - 6) |  | 30 |  | 3 | (2 - 4) |  | 74 |  | 4 | (3 - 5) |
| Wave 3 (mean age 15.9 years) |  | 47 |  | 5 | (4 - 6) |  | 33 |  | 3 | (2 - 4) |  | 80 |  | 4 | (3 - 5) |
| Wave 4 (mean age 16.3 years) |  | 49 |  | 5 | (4 - 7) |  | 54 |  | 5 | (4 - 7) |  | 103 |  | 5 | (4 - 6) |
| Wave 5 (mean age 16.8 years) |  | 78 |  | 8 | (6 - 10) |  | 66 |  | 7 | (5 - 8) |  | 144 |  | 7 | (6 - 9) |
| Wave 6 (mean age 17.4 years) |  | 69 |  | 7 | (5 - 9) |  | 38 |  | 4 | (3 - 5) |  | 107 |  | 6 | (4 - 7) |
|  |  |  |  |  |  |  |  |  |  |  |  |  |  |  |  |
| **by phase** |  |  |  |  |  |  |  |  |  |  |  |  |  |  |  |
| In early adolescence (waves 1&2) |  | 51 |  | 5 | (4 - 7) |  | 37 |  | 4 | (3 - 5) |  | 88 |  | 5 | (4 - 5) |
| In late adolescence (waves 3-6) |  | 168 |  | 18 | (15 - 21) |  | 140 |  | 14 | (12 - 16) |  | 308 |  | 16 | (14 - 18) |
| In adolescence (waves 1-6) |  | 186 |  | 20 | (17 - 23) |  | 157 |  | 16 | (13 - 18) |  | 343 |  | 18 | (16 - 19) |
|  |  |  |  |  |  |  |  |  |  |  |  |  |  |  |  |

**Table S2: Estimates of incident very risky drinking in late adolescence by wave and sex**

| **Incident very risky drinking** |  | **Male** | | | | | |  | **Female** | | | | | |  |  | | **Total** | | | |
| --- | --- | --- | --- | --- | --- | --- | --- | --- | --- | --- | --- | --- | --- | --- | --- | --- | --- | --- | --- | --- | --- |
|  |  | **Total^+^** | | **Number^#^** | | **%** | **(95%CI)** |  | **Total^+^** | | **Number^#^** | | **%** | **(95%CI)** |  | **Total^+^** | | **Number^#^** | | **%** | **(95%CI)** |
| Wave 3 (mean age 15.9 years) |  | 892 |  | 32 |  | 4 | (2 - 5) |  | 963 |  | 21 |  | 2 | (1 - 3) |  | 1855 |  | 53 |  | 3 | (2 - 4) |
| Wave 4 (mean age 16.3 years) |  | 860 |  | 31 |  | 4 | (2 - 5) |  | 942 |  | 42 |  | 4 | (3 - 6) |  | 1803 |  | 72 |  | 4 | (3 - 5) |
| Wave 5 (mean age 16.8 years) |  | 830 |  | 47 |  | 6 | (4 - 7) |  | 901 |  | 39 |  | 4 | (3 - 6) |  | 1730 |  | 85 |  | 5 | (4 - 6) |
| Wave 6 (mean age 17.4 years) |  | 783 |  | 26 |  | 3 | (2 - 5) |  | 862 |  | 19 |  | 2 | (1 - 3) |  | 1645 |  | 45 |  | 3 | (2 - 4) |
|  |  |  |  |  |  |  |  |  |  |  |  |  |  |  |  |  |  |  |  |  |  |

^+^ Total number of adolescents seen at each wave with no previous occurrence of very high risk drinking

^#^ Number of adolescents with incident very high risk drinking at wave

**Table S3: Estimates of the prevalence of repeated (3+ times) drinking in each context in early adolescence by sex**

| **Context of repeated drinking** |  | **Male**  **(N=943)** | | | |  | **Female**  **(N=1000)** | | | |  | **Total**  **(N=1943)** | | | |
| --- | --- | --- | --- | --- | --- | --- | --- | --- | --- | --- | --- | --- | --- | --- | --- |
|  |  | **Number** | | **%** | **(95%CI)** |  | **Number** | | **%** | **(95%CI)** |  | **Number** | | **%** | **(95%CI)** |
| With family |  | 163 |  | 17 | (15 - 20) |  | 138 |  | 14 | (11 - 16) |  | 301 |  | 15 | (14 - 17) |
| Home alone |  | 80 |  | 8 | (6 - 10) |  | 70 |  | 7 | (5 - 9) |  | 149 |  | 8 | (6 - 9) |
| At a pub/club |  | 90 |  | 10 | (8 - 11) |  | 122 |  | 12 | (10 - 14) |  | 212 |  | 11 | (9 - 12) |
| At a party |  | 274 |  | 29 | (26 - 32) |  | 267 |  | 27 | (24 - 30) |  | 541 |  | 28 | (26 - 30) |
| In a park/car |  | 155 |  | 16 | (14 - 19) |  | 148 |  | 15 | (13 - 17) |  | 303 |  | 16 | (14 - 17) |
|  |  |  |  |  |  |  |  |  |  |  |  |  |  |  |  |

**Table S4: Association between context of drinking in early adolescence and incident very risky drinking in later adolescence**

|  | |  | | | **Incidence over waves 3 to 6 (N= 1855)** | | | | | | |
| --- | --- | --- | --- | --- | --- | --- | --- | --- | --- | --- | --- |
|  | | **Number drinking in** | | | **Number incident** | | | **Model estimates^1^** | | | |
|  | | **each joint context^2^** | | | **very risky drinking** | | | **Unadjusted^3^** | | **Adjusted^4^** | |
| **Drinking context** | | **N** | **(%)** |  | | **N** | **(%)** | **RR** | **(95% CI)** | **RR** | **(95% CI)** |
|  |  |  |  |  | |  |  |  |  |  |  |
| With family | *no* | 1585 | (85) |  | | 188 | (12) | 1 |  | 1 |  |
|  | *yes* | 270 | (15) |  | | 67 | (25) | 2.3 | (1.7 - 3.1) | 1.8 | (1.3 - 2.5) |
| Home alone | *no* | 1741 | (94) |  | | 214 | (12) | 1 |  | 1 |  |
|  | *yes* | 114 | (6) |  | | 41 | (36) | 3.5 | (2.4 - 5.2) | 2.1 | (1.4 - 3.2) |
| At a pub/club | *no* | 1690 | (91) |  | | 198 | (12) | 1 |  | 1 |  |
|  | *yes* | 165 | (9) |  | | 57 | (34) | 3.4 | (2.5 - 4.8) | 2.2 | (1.5 - 3.2) |
| At a party | *no* | 1397 | (75) |  | | 111 | (8) | 1 |  | 1 |  |
|  | *yes* | 458 | (25) |  | | 145 | (32) | 4.7 | (3.5 - 6.2) | 3.2 | (2.3 - 4.4) |
| In a park/car | *no* | 1619 | (87) |  | | 167 | (10) | 1 |  | 1 |  |
|  | *yes* | 236 | (13) |  | | 89 | (38) | 4.4 | (3.3 - 5.9) | 2.6 | (1.9 - 3.7) |

1 Separate model fitted for each drinking context

2 Prevalence for those who had no very high risk drinking in waves 1 & 2

3 Relative risks from multivariable discrete time proportional hazards models adjusted for wave of observation of very high risk drinking.

4 Relative risks from multivariable discrete time proportional hazards models adjusted for: (a) the wave of observation of risky drinking, (b) background measures: sex, rural school location, parental separation/divorce, parental frequent alcohol use and parental cigarette smoking (c) adolescent measures from the previous wave: daily cigarette smoking, weekly+ cannabis use, antisocial behaviour and symptoms of anxiety and depression.

**Table S5: Association between joint context of repeated drinking in early adolescence and incident very risky drinking in later adolescence**

| **Joint drinking contexts in early adolescence** | |  | | | **Incidence over waves 3 to 6 (N= 1855)** | | | | | | |
| --- | --- | --- | --- | --- | --- | --- | --- | --- | --- | --- | --- |
| **(waves 1 & 2)** | | **Number drinking in** | | | **Number incident** | | | **Model estimates^1^** | | | |
|  | | **each joint context^2^** | | | **very risky drinking** | | | **Unadjusted^3^** | | **Adjusted^4^** | |
| **Non family context** | **Family context** | **N** | **(%)** |  | | **N** | **(%)** | **RR** | **(95% CI)** | **RR** | **(95% CI)** |
| Not home alone | Not with family | 1531 | (83) |  | | 172 | (11) | 1 |  | 1 |  |
| Not home alone | With family | 210 | (11) |  | | 42 | (20) | 1.9 | (1.3 - 2.8) | 1.5 | (1.00 - 2.3) |
| Home alone | Not with family | 54 | (3) |  | | 16 | (29) | 3.0 | (1.6 - 5.6) | 1.6 | (0.81 - 3.1) |
| Home alone | With family | 60 | (3) |  | | 25 | (42) | 4.7 | (3.0 - 7.6) | 3.1 | (1.8 - 5.2) |
| Not at a party | Not with family | 1267 | (68) |  | | 92 | (7) | 1 |  | 1 |  |
| Not at a party | With family | 130 | (7) |  | | 18 | (14) | 2.0 | (1.1 - 3.5) | 1.7 | (0.97 - 3.0) |
| At a party | Not with family | 318 | (17) |  | | 95 | (30) | 4.8 | (3.4 - 6.7) | 3.2 | (2.2 - 4.7) |
| At a party | With family | 140 | (8) |  | | 49 | (35) | 5.8 | (4.0 - 8.6) | 4.0 | (2.6 - 6.1) |
| Not at pub/club | Not with family | 1486 | (80) |  | | 155 | (10) | 1 |  | 1 |  |
| Not at pub/club | With family | 203 | (11) |  | | 44 | (21) | 2.2 | (1.5 - 3.3) | 1.9 | (1.3 - 2.8) |
| At pub/club | Not with family | 99 | (5) |  | | 33 | (34) | 3.9 | (2.5 - 6.0) | 2.5 | (1.5 - 4.1) |
| At pub/club | With family | 67 | (4) |  | | 24 | (35) | 4.0 | (2.4 - 6.5) | 2.5 | (1.5 - 4.2) |
| Not in park/car | Not with family | 1418 | (76) |  | | 132 | (9) | 1 |  | 1 |  |
| Not in park/car | With family | 200 | (11) |  | | 35 | (17) | 2.0 | (1.3 - 3.0) | 1.7 | (1.1 - 2.5) |
| In park/car | Not with family | 167 | (9) |  | | 56 | (34) | 4.3 | (3.0 - 6.1) | 2.5 | (1.7 - 3.7) |
| In park/car | With family | 70 | (4) |  | | 33 | (47) | 6.6 | (4.2 - 10.5) | 3.8 | (2.3 - 6.4) |
| Not in any other context | Not with family | 1219 | (66) |  | | 82 | (7) | 1 |  | 1 |  |
| Not in any other context | With family | 105 | (6) |  | | 14 | (13) | 2.0 | (1.1 - 3.7) | 1.8 | (0.95 - 3.3) |
| In at least one other context | Not with family | 367 | (20) |  | | 106 | (29) | 5.0 | (3.6 - 6.8) | 3.3 | (2.3 - 4.7) |
| In at least one other context | With family | 165 | (9) |  | | 54 | (33) | 5.8 | (4.0 - 8.4) | 4.0 | (2.7 - 6.0) |

1 Separate model fitted for each joint drinking context

2 Prevalence for those who had no very high risk drinking in waves 1 & 2

3 Relative risks from multivariable discrete time proportional hazards models adjusted for wave of observation of very high risk drinking.

4 Relative risks from multivariable discrete time proportional hazards models adjusted for: (a) the wave of observation of risky drinking, (b) background measures: sex, rural school location, parental separation/divorce, parental frequent alcohol use and parental cigarette smoking (c) adolescent measures from the previous wave: daily cigarette smoking, weekly+ cannabis use, antisocial behaviour and symptoms of anxiety and depression.
